# Risk factors for elevated liver enzymes during refeeding of severely malnourished patients with eating disorders: a retrospective cohort study

**DOI:** 10.1186/s40337-016-0127-x

**Published:** 2016-12-07

**Authors:** Miho Imaeda, Satoshi Tanaka, Hiroshige Fujishiro, Saki Kato, Masatoshi Ishigami, Naoko Kawano, Hiroto Katayama, Kunihiro Kohmura, Masahiko Ando, Kazuo Nishioka, Norio Ozaki

**Affiliations:** 1 Department of Psychiatry, Nagoya University Graduate School of Medicine, 65 Tsurumai, Showa, Nagoya, Aichi 466-8550 Japan; 2 Sakura Clinic, 5-6 Dankeidori, Showa, Nagoya, Aichi 466-0842 Japan; 3 Department of Psychiatry, Nagoya University Hospital, 65 Tsurumai, Showa, Nagoya, Aichi 466-8550 Japan; 4 Saku Central Hospital, 197 Usuda, Saku, Nagano 384-0301 Japan; 5 Department of Gastroenterology and Hepatology, Nagoya University Hospital, 65 Tsurumai, Showa, Nagoya, Aichi 466-8550 Japan; 6 Institute of Innovation for Future Society, Nagoya University, Furocho, Chikusa, Nagoya, Aichi 464-8601 Japan; 7 Department of Neuropsychiatry, Faculty of Medical Sciences, University of Fukui, 23-3, Matsuokashimoaizuki, Eiheiji, Yoshida, Fukui 910-1104 Japan; 8 Seichiryo Hospital, 16-27 Tsurumai 4, Showa, Nagoya, Aichi 466-0064 Japan; 9 Center for Advanced Medicine and Clinical Research, Nagoya University Hospital, 65 Tsurumai, Showa, Nagoya, Aichi 466-8550 Japan; 10 National Hospital Organization Higashi Owari National Hospital, 1301 Omorikita 2, Moriyama, Nagoya, Aichi 463-0802 Japan

**Keywords:** Eating disorder, Anorexia nervosa, Refeeding, Liver injury, Liver enzymes

## Abstract

**Background:**

There are few previous reports regarding the cause and evolution of liver injury in patients with anorexia nervosa (AN) during the refeeding process, and its management remains controversial. This study aimed to determine the risk factors for elevated liver enzymes during refeeding and their effect on the therapeutic process in severely malnourished patients with eating disorders.

**Methods:**

In a retrospective cohort study of 167 female inpatients in a single hospital from January 2004 to March 2015, 67 who had normal alanine aminotransferase (ALT) levels on admission were divided into two groups according to the presence or absence of elevated ALT levels during refeeding, and then compared.

**Results:**

The median age and body mass index (BMI) of the patients on admission were 22 [interquartile range (IQR), 16–33] years and 12.2 (IQR, 11.1–13.0) kg/m^2^, respectively. Compared with their cohorts, significantly more patients in the early onset age group (<15 years old) had elevated ALT levels during refeeding (67% vs. 33%, *p* = 0.033), as did patients with longer median time to nadir BMI (3.0 vs. 0 days, *p* = 0.03). In addition, onset age [odds ratio (OR): 0.274; 95% confidence interval (CI): 0.077–0.981; *p* = 0.047] and time to nadir BMI (OR: 1.271; 95% CI: 1.035–1.56; *p* = 0.022) were significantly associated with the odds of elevated ALT levels during refeeding.

**Conclusions:**

The results of this study suggest that early age at onset may be a potential risk factor for elevated ALT levels during refeeding in severely malnourished patients with eating disorders. Furthermore, elevated ALT levels during refeeding were significantly associated with delay in the start of weight gain. No significant relationship was found between the amount of initial prescribed calories and elevated ALT levels during refeeding. The median time to maximum ALT was 27 (IQR, 21–38) days after the refeeding process started.

**Electronic supplementary material:**

The online version of this article (doi:10.1186/s40337-016-0127-x) contains supplementary material, which is available to authorized users.

## Plain English summary

Refeeding is a therapeutic process for malnourished patients with eating disorders. The development of elevated liver enzymes during this process often becomes a clinical issue. Previous studies report that liver enzymes may become elevated as a result of either the body becoming emaciated or the refeeding process itself. The focus of this study was on the refeeding process. The results suggest that early onset age is a potential risk factor for the development of elevated liver enzymes during refeeding. Furthermore, we found that delay in the start of weight gain may be associated with elevated liver enzymes during refeeding. The amount of initial calorie intake did not have a negative effect on liver dysfunction during refeeding in this study.

## Background

Weight restoration and nutritional rehabilitation are fundamental steps in the treatment program for patients with anorexia nervosa (AN) [1]. The prolonged state of starvation in patients with AN increases cognitive distortion and complicates both commencement of therapy and cooperation during treatment. In addition, the physical condition of patients with AN often deteriorates, leading to a critical illness. The initial and most crucial goal of treatment is to break such patients out of the vicious cycle of weight loss and cognitive distortion by helping them recover weight and improve their nutritional condition [2]. The eventual goals of nutritional rehabilitation for seriously malnourished patients with AN are to restore weight, normalize eating patterns, achieve normal perceptions of hunger and satiety, and correct the biological and psychological sequelae of malnutrition [1]. However, the development of elevated liver enzymes during refeeding often becomes a clinical issue during the therapeutic process. Although rare, there are reports of patients with AN who developed marked fatty liver accumulation, leading to fatal hepatic failure [3], or who developed liver injury with hypoglycemic coma during refeeding [4].

The precise mechanisms involved in the pathogenesis of elevated liver enzymes in patients with AN have yet to be clearly defined. However, recently, a multifactorial etiology has been hypothesized. Hepatocellular injuries associated with non-alcoholic fatty liver disease have been regarded as a cause of elevated liver enzymes in patients with AN and those recovering from malnutrition [3, 5, 6]. Some reports have presented the relationship between low body mass index (BMI) and hepatocellular injuries [7–11]. Suggested etiologies include acute liver hypoperfusion [12, 13], hepatic steatosis with oxidative stress [6], and starvation-induced hepatocyte autophagy [14, 15].

Conversely, mild-to-moderate liver enzyme elevation is common in patients with AN during the refeeding process [7, 16]. Liver enzyme elevation during refeeding may pose a dilemma for clinicians when deciding whether to delay the progressive increase in caloric intake. Progression of refeeding may worsen liver injury, but there is evidence that faster weight gain predicts year 1 weight recovery [17]. To our knowledge, the only published data related to this problem show that the initial prescribed caloric intake may be associated with elevated liver enzymes during refeeding [10]. Little remains known about the causes and optimal management of elevated liver enzymes in clinical practice.

Therefore, the objectives of the present study were to clarify the risk factors for elevated liver enzymes during refeeding in severely malnourished patients with eating disorders and to reveal their effects on the process of nutritional rehabilitation. To achieve these objectives, we retrospectively analyzed clinical variables in a relatively large cohort of severely underweight patients with eating disorders.

## Methods

This study was conducted with the following objectives: 1) to identify potential risk factors associated with the development of elevated liver enzymes during refeeding from among the patients’ background characteristics, blood biochemical findings on admission, and the amount and speed of caloric intake during hospitalization; and 2) to examine whether the development of elevated liver enzymes during refeeding affects the therapeutic course (e.g., duration of hospitalization, ratio of BMI increase, and time to nadir BMI during the hospitalization).

### Study population

This retrospective observation study included 304 patients who were admitted to the Psychiatry Department of Nagoya University Hospital in Nagoya, Japan between 1 January 2004 and 31 March 2015 for nutritional rehabilitation, and who fulfilled the following inclusion criteria: 1) a diagnosis of anorexia nervosa–restricting type (AN-R), anorexia nervosa–binge-eating/purging type, avoidant/restrictive food intake disorder, or other specified feeding or eating disorder, according to the Diagnostic and Statistical Manual of Mental Disorders, Fifth Edition (DSM-5) [18]; 2) a duration of hospitalization longer than 14 days; 3) blood and laboratory data recorded at least twice; and 4) a BMI of less than 15 kg/m^2^ on admission.

Exclusion criteria included the following: 1) male sex, as 97% (295/304) of the patients were female; 2) patients with positive serological tests for hepatitis B or C on admission; 3) patients with a history of hepatic disease or drug or alcohol abuse; and 4) patients with insufficient clinical data. After applying these exclusion criteria, 167 patients were selected. Two psychiatrists (MI and ST) used the DSM-5 criteria to re-diagnose those patients who had been diagnosed before 2013 using the DSM-IV-TR.

### Refeeding protocol

As a rule, nutritional rehabilitation therapy at Nagoya University Hospital was commenced using oral feeding. A high-calorie liquid dietary supplement was administered to replenish the daily caloric intake of patients with insufficient oral intake. Enteral refeeding by nasogastric tube was additionally used for patients who had insufficient weight gain or frequent episodes of hypoglycemia. A number of patients presented with severe malnutrition (BMI <12 kg/m^2^) on admission. In such cases, refeeding was started gradually with the administration of 500–1000 kcal/day, in consideration of the amount of energy intake prior to admission. Refeeding was followed by a progressive increase of 200 kcal every 3 days, according to the patient’s clinical status, with an objective of 1 kg of weight gain per week. In our hospital ward, bringing any medications, such as laxatives, into the room is strictly prohibited, and patients are thoroughly searched. Moreover, patients eat in the day room under supervision and are required to stay there for an hour after eating to control their purging behavior. Fundamentally, nutritional rehabilitation therapy was based on the American Psychiatric Association Practice Guidelines [1]. Twelve of 167 patients received parenteral nutrition because of difficulties with oral or enteral feeding.

### Study design

The demographic and clinical data reported in the present study were obtained retrospectively by electronic chart review for each patient on admission, and included the following: age; age of illness onset; duration of illness; weight; height; blood biochemical data; and the amount of caloric intake during the refeeding process. In our hospital, height is measured on admission. Weight is also measured on admission and twice a week using a strict protocol: under supervision, patients are required to urinate or defecate, change into a hospital gown, and step onto a scale after a body check to confirm that they do not have anything to falsify their body weight. In the case of patients who drank an excessive amount of water, we also examine laboratory data around the same time to confirm the presence of overhydration [suggested by the presence of dilutional hyponatremia, dilutional anemia, and low blood urea nitrogen (BUN) level]. If patients were admitted to Nagoya University Hospital more than once, data from the first hospitalization were used. Medical charts were reviewed independently by a psychiatrist (MI) and a dietician (SK), and a further evaluation of clinical parameters, including diagnosis and age of illness onset, was conducted by two psychiatrists (MI and ST) experienced in treating eating disorders.

Clinical parameters analyzed included the following: duration of hospitalization; BMI on admission; BMI on discharge; ratio of BMI increase (BMI on discharge/BMI on admission); speed of BMI increase, calculated as [(BMI on discharge – BMI on admission)/duration of hospitalization]; nadir BMI during hospitalization; time to nadir BMI; initial prescribed calories/kg; maximum prescribed calories/kg; and speed of caloric increase, calculated as [(maximum prescribed calories – initial prescribed calories)/days to maximum prescribed calories]. As a rule, we examine the first serum chemistries before refeeding on admission. After refeeding therapy is started, blood is drawn every day or every third day as clinically indicated. When the general condition of the patients is stabilized, blood is drawn once a week during hospitalization. Routine laboratory data obtained on admission included the following: total protein (TP); albumin; glucose; BUN; creatinine; aspartate aminotransferase (AST); alanine aminotransferase (ALT); lactate dehydrogenase; alkaline phosphatase; gamma glutamyl transferase; white blood cells; red blood cells; hemoglobin, hematocrit; and platelets. BMI was calculated as weight in kilograms divided by the square of height in meters (kg/m^2^).

ALT is a specific measure of liver injury [19], so we determined that any elevation in ALT from baseline was a sign of liver injury, and regarded it as a symptom of liver damage. ALT >27 IU/L was considered elevated according to the upper limit of the normal level used by the Nagoya University Hospital laboratory.

**Fig. 1:**
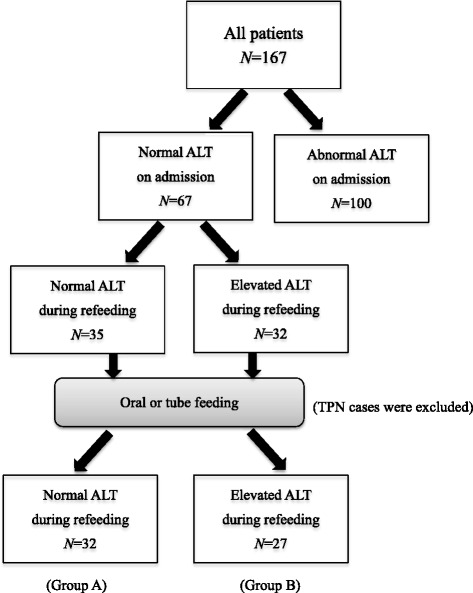
Enrollment flow chart. ALT, alanine aminotransferase; TPN, total parenteral nutrition

First, the enrolled patients were divided into two groups according to the presence or absence of elevated ALT levels on admission (Fig. 1). Next, we selected the 67 patients who had normal ALT levels on admission for inclusion in the study, and excluded those with elevated ALT levels on admission, which was probably associated with starvation before refeeding. The 67 patients with normal ALT levels on admission were then categorized into the following two groups: 1) Group A, who had normal ALT levels during refeeding (*N* = 35); and 2) Group B, who had elevated ALT levels during refeeding (*N* = 32). Eight patients (three from Group A and five from Group B) who received total parenteral nutrition (TPN) were excluded because TPN was started later as the need arose, and because it involves different metabolic pathways than oral or enteral feeding. Finally, 59 patients receiving oral and/or tube feeding comprised the final groups, categorized as Group A (normal ALT levels during refeeding; *N* = 32) and Group B (elevated ALT levels during refeeding; *N* = 27). Patients in Group A had normal ALT levels throughout hospitalization, while patients in Group B presented with normal ALT levels on admission and then developed elevated ALT levels during refeeding. Groups A and B were then compared using the previously described clinical parameters.

### Statistical analysis

Univariate statistics were used to describe the sample, including frequency (%), range, and median [interquartile range (IQR)]. We used Mann-Whitney *U* tests to compare the medians of continuous variables (such as age) and chi-square tests to compare the proportions of categorical variables (such as onset age) between groups. Multivariate analysis using logistic regression was performed with the presence of elevated ALT levels during refeeding as the dependent variable. The independent variables included BMI on admission, initial prescribed calories (which was considered clinically important in a previous report) [10], and factors identified by univariate analysis. Values of *p* < 0.05 were considered statistically significant. All statistical analyses were performed using the Statistical Package for Social Sciences (SPSS) (ver. 23, IBM, Armonk, NY).

## Results

### Patients’ characteristics

During the 11 years and 3 months of the study period, 304 patients with eating disorders from 500 total hospitalizations were admitted to the Psychiatry Department of Nagoya University Hospital, 65% of whom were admitted only once. After applying the exclusion criteria, 167 patients were eligible for inclusion in this study. The patients had a median age of 22 (IQR, 16–33) years and a median illness onset age of 17 (IQR, 14–20) years (Table 1). The median BMI on admission was 12.2 (IQR, 11.1–13.0) kg/m^2^, and the median amount of initial prescribed calories was 37.1 (IQR, 27.7–50.3) kcal/kg. In total, 92% (154/167) of the patients were diagnosed with AN, and more than half (55%; 92/167) were diagnosed with AN-R. Based on category definitions used in previous reports [20, 21], of the 167 patients, 28% (*N* = 47) were in the early onset age (<15 years old) group; 60% (*N* = 100) were in the peak onset age (15–24 years old) group; and 12% (*N* = 20) were in the late onset age (≥25 years old) group. The median duration of hospitalization was 58 (IQR, 36–82) days, with the longest stay being 285 days.

**Table 1:** Patients’ characteristics (*N* = 167)

	Median (IQR)	Range
Age (years)	22 (16–33)	10–54
Age of illness onset (years)	17 (14–20)	8–45
Duration of illness (years)	2.2 (1.0–11.0)	0.2–40.0
Duration of hospitalization (days)	58 (36–82)	15–285
BMI on admission (kg/m^2^)	12.2 (11.1–13.0)	8.1–14.9
BMI on discharge (kg/m^2^)	14.6 (13.9–15.3)	11.3–18.5
Ratio of BMI increase^a^	1.2 (1.1–1.3)	1.0–1.8
Speed of BMI increase ([kg/m^2^]/days)^b^	0.04 (0.03–0.05)	0–0.13
Minimum BMI during hospitalization (kg/m^2^)	11.9 (10.8–12.8)	8.1–14.6
Time to nadir BMI (days)	2 (0–6)	0–43
Initial prescribed calories (kcal/kg)	37.1 (27.7–50.3)	5.9–115.3
Maximum prescribed calories (kcal/kg)	69.6 (57.3–80.7)	29.5–125.0
Speed of caloric increase (kcal/days)^c^	35.6 (19.2–54.7)	0–595.0
	Frequency (%)	
AN-R	92 (55)	
AN-BP	62 (37)	
ARFID	8 (5)	
OSFED	5 (3)	
Early onset (<15 years old)	47 (28)	
Peak onset (15–24 years old)	100 (60)	
Late onset (≥25 years old)	20 (12)	

*IQR* interquartile range, *BMI* body mass index, *AN-R* anorexia nervosa–restricting type, *AN-BP* anorexia nervosa–binge-eating/purging type, *ARFID* avoidant/restrictive food intake disorder, *OSFED* other specified feeding or eating disorder, *BW* body weight

^a^Ratio of BMI increase, BMI on discharge/BMI on admission; ^b^Speed of BMI increase: (BMI on discharge–BMI on admission)/duration of hospitalization; ^c^Speed of caloric increase: [(maximum prescribed calories–initial prescribed calories)]/days to maximum prescribed calories

Blood biochemical findings of all enrolled patients on admission are shown in Table 2. A total of 100 (60%) patients had elevated ALT levels on admission (Fig. 1), while 132 (79%) had elevated ALT levels at some point during hospitalization. Of the 67 patients with normal ALT levels on admission, 32 (48%) developed elevated ALT levels during refeeding.

**Table 2:** Blood biochemical findings on admission (*N* = 167)

	Median (IQR)	Range
TP (6.7–8.3 g/dL)^a^	6.6 (6.1–7.1)	4.3–8.4
Albumin (4–5 g/dL)	4.2 (3.9–4.7)	1.9–5.5
Glucose (70–109 mg/dL)	76 (67–88)	20–166
BUN (8–22 mg/dL)	15 (11–23)	4–60
Creatinine (0.4–0.7 mg/dL)	0.6 (0.5–0.7)	0.2–1.5
AST (13–33 IU/L)	32 (23–62)	5–2881
ALT (6–27 IU/L)	33 (20–86)	6–2751
LDH (119–229 IU/L)	227 (184–304)	112–746
ALP (115–359 IU/L)	193 (140–263)	24–1163
GGT (10–47 IU/L)	29 (16–57)	8–423
WBC (3.8–8.5 × 10^3^/μL)	3.8 (3.0–4.7)	1.2–14.1
RBC (3.6–5.0 × 10^6^/μL)	3.98 (3.59–4.32)	2.02–5.53
Hemoglobin (11–16 g/dL)	12.3 (11.4–13.3)	7.4–17.9
Hematocrit (32–48%)	36.5 (33.9–39.5)	21.5–47.3
Platelets (160–410 × 10^3^/μL)	201 (156–245)	34–566

*IQR* interquartile range, *TP* total protein, *BUN* blood urea nitrogen, *AST* aspartate aminotransferase, *ALT* alanine aminotransferase, *LDH* lactate dehydrogenase, *ALP* alkaline phosphatase, *GGT* gamma glutamyl transferase, *WBC* white blood cells, *RBC* red blood cells

^a^Reference range shown in parentheses

**Fig. 2:**
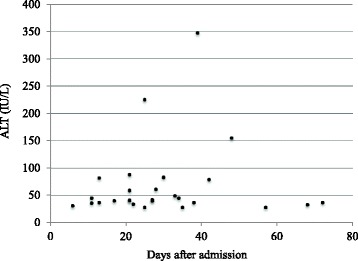
Timing and magnitude of maximum ALT levels for patients in Group B (*N* = 27). ALT, alanine aminotransferase. Patients in Group B had elevated ALT levels (>27 IU/L) during refeeding

Maximum ALT levels of the patients in Group B ranged over 28–347 IU/L, with a median of 41 (IQR, 36–79) IU/L, indicating mild-to-moderate elevation [19]. The median time to maximum ALT in Group B was 27 (IQR, 21–38) days, with the longest time being 72 days (Fig. 2). Elevated ALT levels in Group B peaked 27 days after the refeeding process started.

Among the patients in Group B, 81% (22 of 27) had follow-up ALT data showing a decrease to the normal range after approximately 1 month [median, 28 (IQR, 10–54) days] from the date of maximum ALT. On the other hand, 7% (2 of 27) had continued ALT elevation after 1 month from the date of maximum ALT (data not shown).

### Comparison of groups A and B

Results from a comparison of Groups A and B are shown in Tables 3 and 4. The number of patients in the early onset age group was significantly higher in Group B than in Group A. The number of patients in the peak onset and late onset groups was significantly higher in Group A than in Group B (χ^2^_(1)_ = 4.56, *p* = 0.033, φ = 0.28). The median time to nadir BMI was significantly longer in Group B than in Group A (3 days vs. 0 days, *U* = 302.5, *p* = 0.03, *r* = 0.28). No significant differences were found between the two groups in age, BMI on admission, duration of illness, or initial prescribed calories. TP was significantly lower in Group B than in Group A (6.6 vs. 7.1 g/dL, *U* = 293, *p* = 0.034, *r* = 0.28).

**Table 3:** Comparison of Group A and Group B (patients’ characteristics)

	Group A		Group B		
	*N* = 32		*N* = 27		
	Median (IQR)	Range	Median (IQR)	Range	*p* value^a^
Age (years)	19 (16–30)	11–46	30 (15–37)	10–54	0.322
Age of illness onset (years)	16 (15–18)	11–35	17 (13–21)	8–38	0.957
Duration of illness (years)	1.6 (0.8–12.5)	0.2–28.0	9.0 (1.0–16.0)	0.5–40.0	0.132
Duration of hospitalization (days)	46 (29–76)	15–138	48 (34–75)	23–154	0.568
BMI on admission (kg/m^2^)	12.2 (11.1–13.3)	9.2–14.3	12.3 (10.8–13.2)	8.1–14.9	0.831
BMI on discharge (kg/m^2^)	14.6 (13.6–15.0)	11.3–18.5	14.6 (13.9–16.1)	11.4–17.4	0.548
Ratio of BMI increase^c^	1.2 (1.1–1.3)	1.0–1.5	1.2 (1.1–1.3)	1.1–1.6	0.389
Speed of BMI increase ([kg/m^2^]/days)^d^	0.04 (0.03–0.05)	0.02–0.13	0.05 (0.03–0.07)	0.01–0.13	0.159
Minimum BMI during hospitalization (kg/m^2^)	12.0 (10.9–12.8)	9.2–14.0	12.2 (10.8–13.2)	8.1–14.4	0.749
Time to nadir BMI (days)	0 (0–1.8)	0–10.0	3.0 (0–6.0)	0–41.0	0.03
Initial prescribed calories (kcal/kg)	37.0 (29.7–47.2)	6.6–77.2	33.2 (27.4–48.2)	16.7–115.3	0.67
Maximum prescribed calories (kcal/kg)	71.8 (56.5–82.8)	47.1–113.1	67.2 (58.5–82.7)	43.0–116.5	0.927
Speed of caloric increase (kcal/days)^e^	36.6 (29.4–59.6)	0–98.3	33.3 (14.6–63.8)	0–595.0	0.721
	Frequency (%)				*p* value^b^
Early onset (<15 years old)	6 (33)		12 (67)		0.033
Peak or late onset (≥15 years old)	26 (63)		15 (37)		

Patients in Group A had normal ALT levels (≤27 IU/L) during refeeding, while patients in Group B had elevated ALT levels (>27 IU/L) during refeeding

*ALT* alanine aminotransferase, *IQR* interquartile range, *BMI* body mass index, *BW* body weight

^c^Ratio of BMI increase: [BMI on discharge/BMI on admission]; ^d^Speed of BMI increase: [(BMI on discharge–BMI on admission)/duration of hospitalization]; ^e^Speed of caloric increase: [(maximum prescribed calories–initial prescribed calories)/days to maximum prescribed calories]; ^a^Mann-Whitney *U* test; ^b^Pearson chi-square test

**Table 4:** Comparison of Group A and Group B (blood biochemical findings on admission)

	Group A		Group B		
	*N* = 32		*N* = 27		
	Median (IQR)	Range	Median (IQR)	Range	*p* value^b^
TP (6.7–8.3 g/dL)^a^	7.1 (6.8–7.5)	5.2–8.3	6.6 (6.4–7.1)	5.9–7.9	0.034
Albumin (4–5 g/dL)	4.6 (3.9–4.9)	2.1–5.3	4.3 (3.7–4.7)	2.7–5.3	0.125
Glucose (70–109 mg/dL)	78 (71–92)	34–132	83 (71–93)	51–121	0.558
BUN (8–22 mg/dL)	14 (12–17)	6–34	13 (9–15)	6–39	0.138
Creatinine (0.4–0.7 mg/dL)	0.64 (0.60–0.70)	0.48–1.24	0.62 (0.54–0.71)	0.26–1.35	0.398
AST (13–33 IU/L)	20 (18–25)	13–63	25 (22–31)	18–56	0.004
LDH (119–229 IU/L)	189 (160–211)	146–272	196 (157–242)	129–370	0.659
ALP (115–359 IU/L)	179 (130–224)	74–514	195 (141–241)	24–758	0.518
GGT (10–47 IU/L)	14 (11–27)	8–109	19 (14–35)	8–120	0.198
WBC (3.8–8.5 × 10^3^/μL)	4.5 (3.8–5.0)	2.4–6.6	3.8 (3.1–5.2)	2.1–9.6	0.161
RBC (3.6–5 × 10^6^/μL)	4.1 (3.8–4.4)	2.6–5.4	3.9 (3.5–4.2)	3.1–5.5	0.128
Hemoglobin (11–16 g/dL)	12.3 (11.5–13.4)	8.1–16.4	11.9 (11.1–13.1)	9.4–16.1	0.349
Hematocrit (32–48%)	36.7 (35.0–40.1)	24.5–47.3	35.8 (33.1–38.6)	28.9–45.5	0.283
Platelets (160–410 × 10^3^/μL)	216 (188–254)	154–396	207 (175–260)	150–490	0.732

Patients in Group A had normal ALT levels (≤27 IU/L) during refeeding, while patients in Group B had elevated ALT levels (>27 IU/L) during refeeding

*ALT* alanine aminotransferase, *IQR* interquartile range, *TP* total protein, *BUN* blood urea nitrogen, *AST* aspartate aminotransferase, *LDH* lactate dehydrogenase, *ALP* alkaline phosphatase, *GGT* gamma glutamyl transferase, *WBC* white blood cells, *RBC* red blood cells

^a^Reference range shown in parentheses. ^b^Mann-Whitney *U* test

### Risk factors related to elevated ALT levels during refeeding

To identify the most important risk factors for elevated ALT levels during refeeding, multivariate analysis using logistic regression was performed with the following four factors (Table 5): onset age; BMI on admission; time to nadir BMI; and initial prescribed calories. As for onset age, a dummy-coded variable (early onset age group = 0, peak or late onset age group = 1) was used. Onset age [odds ratio (OR): 0.274; 95% confidence interval (CI): 0.077–0.981; *p* = 0.047] and time to nadir BMI (OR: 1.271; 95% CI: 1.035–1.56; *p* = 0.022) were significantly associated with the risk of elevated ALT levels during refeeding.

**Table 5:** ORs for determinants of normal or elevated ALT levels during refeeding

	Multivariate	
	OR (95% CI)	*p* value
Onset age	0.274 (0.077–0.981)	0.047
BMI on admission (kg/m^2^)	0.857 (0.578–1.271)	0.442
Time to nadir BMI (days)	1.271 (1.035–1.56)	0.022
Initial prescribed calories (kcal/kg)	1.001 (0.969–1.034)	0.961

*OR* odds ratio, *CI* confidence interval, *ALT* alanine aminotransferase, *BMI* body mass index

## Discussion

In the present study, we examined the risk factors for elevated liver enzymes during refeeding in severely malnourished patients with eating disorders, as well as the effects on the nutritional rehabilitation process. We found that early onset was significantly associated with the development of elevated ALT levels during refeeding. Time to nadir BMI was also significantly associated with elevated ALT levels during refeeding (Tables 3 and Table 5). However, no associations were found between elevated ALT levels during refeeding and variations in the refeeding method, including amount of initial prescribed calories, maximum prescribed calories, and speed of caloric increase (Table 3).

### Early onset age as a risk factor for ALT elevation

To our knowledge, this is the first study to report that early illness onset may be a risk factor for elevated ALT levels during refeeding. One previous study that examined predictors of elevated liver enzymes after admission in patients with AN did not mention the involvement of onset age [10]. The basal metabolic rate among females is highest in the 12–14-year-old age group (1410 kcal/day), and gradually declines with age [22]. Moreover, younger patients with AN may suffer more metabolic damage from nutritional deficiencies [8] or have increased susceptibility to liver damage [23]. The liver plays a central role in regulating energy homeostasis through glucose, lipid, and protein metabolism [24]. Early onset of AN is likely to damage the liver through nutrient deficiencies during the growth process, resulting in some degree of vulnerability thereafter.

According to a previous report, some children with non-alcoholic fatty liver disease are at risk for developing nonalcoholic steatohepatitis [25]. Although there is a difference in clinical conditions between fatty liver caused by starvation and that by obesity, a similar pathogenetic mechanism is hypothesized [24]. Therefore, we think it is reasonable to be concerned with the long-term liver pathology associated with early onset AN.

### Concerning methods of refeeding

In the present study, no significant relationship was found between the amount of initial prescribed calories and elevated ALT levels. This result was different from those of a previous report showing an association between initial prescribed calories and elevated liver enzymes after admission [10]. That study used regression analysis that included age, % median BMI on admission, and duration of illness as variables. We speculate that the difference may be attributable to the fact that the patients in the previous study were younger than those in the present study [mean ± standard deviation, 16.1 ± 2.4 years vs. median, 22 (IQR, 16–33) years, respectively], and the amount of initial prescribed calories was larger [mean, 1452.4 kcal vs. median, 1040 (IQR, 750–1600) kcal, respectively]. Therefore, both younger age and higher initial prescribed calories may be risk factors for elevated liver enzymes in patients with AN. Another possible reason for this difference is that we reduced the initial prescribed calories because our participants had lower BMIs on admission than patients in the previous study [median, 12.2 (IQR, 11.1–13.0) kg/m^2^ vs. mean, 15.9 ± 1.9 kg/m^2^], as well as more severe emaciation with more serious general physical deterioration.

The present study showed that 46% (27/59) of the patients who had normal ALT levels on admission developed elevated ALT levels during refeeding. Conversely, an earlier study showed that patients who did not have hyperaminotransferasemia on admission retained normal liver function during 4 weeks of refeeding [9]. One reason for this might be that the amount of initial prescribed calories in that study was lower than that in the present study (maximum, 30 kcal/kg vs. median, 37.1 kcal/kg); in addition, those patients had less liver damage. Another possibility is that the researchers only considered that there was significant hepatic cytolysis when AST and/or ALT levels were more than twice the upper limit of the normal value. The different cutoff points used might account for these mixed results. Since few studies on liver dysfunction in patients with AN during refeeding have been conducted, further studies will be needed to adequately address this issue.

As previously mentioned, none of the nutritional rehabilitation methods in the present study were associated with elevated liver enzymes during refeeding. The median amount of initial prescribed calories was 37.1 (IQR, 27.7–50.3) kcal/kg, and the median amount of maximum prescribed calories was 69.6 (IQR, 57.3–80.7) kcal/kg. We could not investigate alteration of refeeding in this study. We can only say, therefore, that the volume of caloric intake within these ranges did not have a negative effect on liver function. There are few reports regarding the effect of refeeding strategy on liver injury in patients with AN and further investigation is needed. Moreover, it appears that matters such as inherent characteristic traits related to liver injury and liver vulnerability caused by early age of illness onset before the start of refeeding therapy should be carefully considered.

### Elevated ALT levels may relate to delay in the start of weight gain

In the present study, we found that elevated ALT levels during refeeding were significantly associated with delay in the start of weight gain; to our knowledge, this is the first study to report this finding. We considered the following three possible explanations.

First, the central feature of protein-energy malnutrition such as kwashiorkor and marasmus malnutrition, like AN, is edema [26]. Patients who had normal ALT levels on admission and elevated ALT levels during refeeding, and those who had a trend of lower TP on admission, may have had more clinically severe edema than those who had normal ALT levels throughout their hospitalization. It is therefore possible that after refeeding started, patients with elevated ALT levels had increased urination, which led to a decrease in body weight and delay in the start of weight gain. A previous study showed that refeeding edema developed more frequently in patients with severely elevated liver enzymes [11]. There may be a decrease in liver protein synthesis associated with edema. If we assume the presence of edema in our cohort, we can hypothesize that weight loss at the initial stage of refeeding after prolonged starvation might predict elevation of liver enzymes in the later stage.

A second possibility is that metabolic derangements can occur during the process of refeeding in at-risk patients [27]. Weight gain is not expected to occur during the first 5–7 days of refeeding because this period, termed the “phase of stabilization”, is the period during which a change from a catabolic to an anabolic state occurs [28]. Delay in the start of weight gain among patients with elevated ALT levels during refeeding might reflect the fact that this metabolic change imposes a strain on the entire body, including the liver; given our findings relevant to the association between delay in the start of weight gain and subsequent ALT elevation, it could be assumed that delay in the start of weight gain may be a risk factor for elevated liver enzymes during refeeding.

Finally, it is possible that the amount of caloric intake was less than that required for weight gain, which led to a decrease in weight. Prolonged starvation might cause an emaciation-induced elevation in aminotransferase [7]. Therefore, weight gain or loss during initial nutritional therapy may help distinguish elevated liver enzymes related to refeeding from those related to a state of starvation [16]. Among patients with elevated ALT levels during refeeding, most (25 of 27) had elevated liver enzymes while gaining weight, so it is unlikely that prolonged starvation caused the elevation of liver enzymes.

Based on these findings, it is reasonable to assume that several factors triggered by nutritional rehabilitation contributed to the development of elevated liver enzymes.

### Liver injury during refeeding

Similar to a previous study that showed refeeding-induced aminotransferase elevation [7], the present study found that patients with elevated liver enzymes during refeeding had mild-to-moderate ALT elevations that peaked around the 27^th^ day of hospitalization (Fig. 2). This is consistent with previous findings showing that the period from the nadir of body weight to the peak of the aminotransferase elevation was about 20 days, and that the elevated ALT levels during refeeding was milder than that of the emaciation-induced type, accompanied by higher body weight and better physical condition [7]. The reason why the number of days required to reach maximum ALT was greater in our study than in the previous study may be that while we counted from the first day of hospitalization to the point of peak ALT, the previous results were calculated from the point of nadir body weight to the point of peak ALT. Taken as a whole, the finding of elevated liver enzymes among the patients in Group B during refeeding was similar to that of the previous study. Several hypotheses have been suggested concerning the etiology of these abnormalities, including excessive dextrose calories leading to fat accumulation in the liver cells (hepatic steatosis) [27, 29, 30].

Among the patients in Group B, 81% (22 of 27) had follow-up ALT data showing a decrease to the normal range after approximately 1 month [median, 28 (IQR, 10–54) days] from the date of maximum ALT (data not shown). It can therefore be said that the evolution of elevated ALT levels was favorable in the same way as that observed in TPN-related steatosis [31]. On the other hand, the follow-up ALT data showed that 7% (2 of 27) had continued ALT elevation after 1 month from the date of maximum ALT. There is a case report of a patient with AN who had a long duration of illness and developed malnutrition-related nonalcoholic steatohepatitis [32]. As far as we know, there is no study on the long-term prognosis of liver injury in patients with AN. This issue deserves further research.

### Limitations

The major limitations of this study are its retrospective nature and its inability to prove causality. This study is based on medical chart review, and we were unable to review the data from physical examinations, including the degree of edema. Furthermore, our study sample was limited to severe and hospitalized cases; therefore, the present findings cannot be generalized to outpatient or community samples. Another limitation of the study is that it did not evaluate liver histopathology or morphology using ultrasonography, computed tomography, and magnetic resonance imaging as the basis for diagnosis.

Nutritional rehabilitation was performed under supervision in our structured ward; however, we cannot say that purging behaviors were controlled perfectly. There may have been a difference between the actual amount of consumed calories and the administered amount in some patients. As for body weight measurement, we could not exclude the data of possibly overhydrated patients. Therefore, the present results include some influence from temporary fluctuations in body weight.

In addition, we were unable to exclude patients with elevated liver enzymes who were receiving medication. Nineteen of 32 patients in Group A and 19 of 27 patients in Group B were taking medications, including antidepressants, major and minor tranquilizers, and hypnotics. In multivariate analysis, we added medication use as an independent variable and confirmed that the results for both groups were identical; onset age (OR: 0.151; 95% CI: 0.033–0.697; *p* = 0.015) and time to nadir BMI (OR: 1.296; 95% CI: 1.042–1.613; *p* = 0.02) were significantly associated with the risk of elevated ALT levels during refeeding. Further research using a protocol in which the effect of medication is excluded would be useful.

## Conclusions

In the present study, we aimed to identify the risk factors for elevated liver enzymes during refeeding in severely underweight patients with eating disorders, as well as the effects on the nutritional rehabilitation process. We found that an early age of onset was significantly associated with the development of elevated ALT levels during refeeding. No associations were found between elevated ALT levels during refeeding and variations in the refeeding method, including initial prescribed calories, maximum prescribed calories, and speed of caloric increase. However, a significant relationship was found between elevated ALT levels during refeeding and time to nadir BMI; i.e., elevated ALT levels during refeeding was significantly associated with delay in the start of weight gain. The median time to maximum ALT was 27 (IQR, 21–38) days after the refeeding process started.

Little previous research has been conducted concerning liver enzyme elevation during refeeding in patients with AN, and consensus regarding the pathophysiological mechanisms involved or the best management approach has yet to be reached. It is hoped that the results of this exploratory study will lead to more definitive results from prospective studies in the future.

## Additional file

Additional file 1: Raw Data Table. (XLSX 133 kb)

## Abbreviations

ALT: Alanine aminotransferase
AN: Anorexia nervosa
AN-R: Anorexia nervosa–restricting type
BMI: Body mass index
BUN: Blood urea nitrogen
CI: Confidence interval
DSM-5: Diagnostic and Statistical Manual of Mental Disorders, Fifth Edition
IQR: Interquartile range
OR: Odds ratio
TP: Total protein
TPN: Total parenteral nutrition
